# Federated training of spiking neural networks on edge hardware for audio processing

**DOI:** 10.3389/fnins.2026.1827009

**Published:** 2026-05-13

**Authors:** Swaroop S. Kaimal, Ashwin JB, S. Sofana Reka, Prakash Venugopal

**Affiliations:** 1 School of Electronics Engineering, Vellore Institute of Technology, Chennai, India; 2 Centre for Neuroinformatics, School of Electronics Engineering, Vellore Institute of Technology, Chennai, India

**Keywords:** brain-inspired computing, edge computing, federated training, signal processing, spiking neural networks (SNNs), neural networks, internet of things, deep learning

## Abstract

Spiking Neural Networks have caught significant attention recently for their potential for energy-efficient computation on neuromorphic hardware and their event-driven processing. Spiking Neural networks employ spike-based learning paradigms, which require specialized training procedures such as Surrogate Gradient Descent. At the same time, Federated Learning allows collaborative model training on decentralized devices with preservation of data privacy protection. However, to date, few research has examined the suitability of Federated learning with ARM-based hardware. This work primarily investigates whether Federated Spiking Neural Networks training on ARM-based hardware is feasible with the Raspberry Pi 5 as a widely available and low-cost edge computing device for audio signal processing tasks. We perform a comparative analysis of federated Spiking Neural Network and federated convolutional neural networks on ARM processors and evaluate their performance on different data partitioning strategies using Dirichlet-based splits and various federated averaging algorithms. Using Federated learning, this work investigates the impact of data heterogeneity and aggregation strategies on model convergence, communication overhead, and latency in distributed training paradigms. The results provided showcases the important insights into the trade-offs of FL-SNN implementations on Von Neumann architectures and their applications in decentralized neuromorphic computing for audio processing.

## 1 Introduction

The rapid growth of edge computing has led to a wide distribution of needs for power-efficient and privacy-preserving machine learning models, particularly for applications on embedded systems and edge devices (Fanariotis et al., 2023). Deep learning models are highly efficient but are also incredibly power-consuming and computationally intensive and in general are not highly reliable for real-time deployment in edge computing systems (Elhanashi et al., 2024). Deployment in such systems is inefficient for edge audio processing systems where activities such as audio classification and real-time speech recognition require continuous learning with low latency and low power consumption (Basak et al., 2023). Spiking Neural Networks (SNNs) are an alternative to the widely well-established deep learning models since they are event driven and designed for low-energy operation on neuromorphic hardware while maintaining the performance of sequential data processing (Tavanaei et al., 2019). Unlike conventional Artificial Neural Networks (ANNs), SNNs transmit data in a discrete form, in the form of spikes which is significantly comparable to the neural function of biological neurons. This makes SNNs especially well-suited for edge computing operations where neuromorphic hardware can exploit their spike-based sparsity for energy-efficient inference (Nunes et al., 2022). However, training of SNNs remains one of the most challenging problems, especially in decentralized or distributed re-use scenarios, which take place over data produced at the edge device at a local level. Federated Learning (FL) is a decentralized learning technique that allows for collaborative learning across devices and to maintain the raw data on the devices with the aim of preserving privacy and security (Martínez Beltrán et al., 2023). FL is applied to privacy-critical workloads such as voice recognition, where the transmission of raw speech data to the central server is confidential and security impeded (Pelikan et al., 2023). It has the benefit of allowing the central server to receive global model updates without disclosing raw data, after which edge devices can compute models locally (Nguyen et al., 2021). Distributed federated learning, however, has not been widely explored on SNNs, especially on Von Neumann-based systems. Communication overhead has been identified as one of the major disadvantages in federated training of neural net- works. Federated learning involves overlapping communication operations between the central server and edge devices, which increases network latency and energy consumption and makes large-scale network deployment challenging (Shahid et al., 2021). Non-independent and identically distributed (non-IID) data on edge devices is another issue. Centralized training involves gathering device data in a manner that maximizes the model’s performance. Local data is still linked to individual devices in federated learning, resulting in a distinct data distribution that also helps to avoid generalization and model convergence (Lu et al., 2024; Manna et al., 2022).

Training SNN on edge devices may demand large computational capabilities especially on non-neuromorphic hardware. Biologically realistic learning rules like Spike-timing-dependent Plasticity (SPDP) and Surrogate Gradient based backpropagation are used in many SNN training protocols (Liu et al., 2021; Neftci et al., 2019). Most Federated Learning (FL) systems assume the utilization of high-end GPUs and access to advanced cloud computing resources. Therefore, it is necessary to develop lightweight and hardware conscious FL methods for SNNs that can be conveniently implemented in a real-world application (Nguyen et al., 2021). Cognitive audio processing at the network edge with competitive accuracy can be made possible by such an implementation of FL-SNN, notwithstanding the previously mentioned restrictions. Prior studies have concentrated on developing real-time, tractable spike-based computation models that may be accessed through neuromorphic hardware-centric computations, enabling inferences on edge setup (Shrestha et al., 2022). We put into practice an end-to-end study of FL-SNN training. To reduce the cost of optimization, we use a laptop as the model aggregation and coordination server and construct the snnTorch framework, which is specifically designed for SNNs, and a surrogate gradient-based optimization method (Eshraghian et al., 2023). Utilizing Dirichlet-based partitions and different federated aver-aging methods, we present a comparative analysis of Federated SNNs and Federated CNNs executed on the ARM processor, providing their performance on different data partitioning methods. Under distributed training, federated learning is used to explore the effect of data heterogeneity and aggregation methods on model convergence rates, accuracy and reliability.

While prior work such as Venkatesha et al. (2021) established the theoretical basis for federated SNN training using BNTT on CIFAR-10 and CIFAR-100 with up to 100 simulated GPU-based clients and FedAvg aggregation, and Xie et al. (2022) demonstrated FL-SNN for traffic sign recognition on the Internet of Vehicles using neuronal receptive field encoding, again on GPU hardware with FedAvg alone, no prior study has: (1) evaluated FL-SNN feasibility on CPU-only ARM-based edge hardware, where the computational constraints differ fundamentally from GPU simulation; (2) conducted a systematic comparison of multiple federated aggregation algorithms (FedAvg, FedAdam, FedPAQ, Top-K FedAvg) specifically for SNN training under Dirichlet-distributed non-IID conditions; (3) characterised the differential behavioural response of SNNs versus CNNs to increasing data heterogeneity in a federated setting; or (4) applied federated SNN training to audio signal processing, a domain where the temporal dynamics of spiking neurons align naturally with the sequential and event-driven characteristics of acoustic data. This paper addresses all four gaps using Raspberry Pi 5 devices as representative low-cost edge nodes, providing the first empirical evidence of how FL-SNN dynamics differ from FL-CNN dynamics on conventional non neuromorphic hardware for respiratory sound classification.

Beyond the independent motivations for SNNs and federated learning, their combination introduces distinct dynamics that merit investigation. SNN weight updates, driven by surrogate gradient approximations of discrete spike events, produce inherently noisier gradient signals than conventional CNNs. When combined with non-IID data in a federated setting, this noise compounds with statistical heterogeneity across clients, making the choice of aggregation strategy potentially more consequential for SNNs than for conventional architectures. Conversely, the natural sparsity of spike-based updates may benefit communication efficiency, as SNN weight deltas are likely to contain greater redundancy than their dense ANN counterparts, enabling more aggressive compression with less information loss. This work investigates both dynamics empirically. In Section 2, prior research on FL, SNNs, and audio processing is reviewed. The suggested FL-SNN model, design, approach, and techniques are presented in Section 3. The experimental design, execution, and results are discussed and analysed in Section 4. The investigation and conclusion are summarized in Section 5, along with suggestions for further research.

## 2 Background

This section provides a brief background of SNNs, Federated Learning, audio signal processing on edge devices and the identified research methods.

### 2.1 Spiking neural networks (SNNs)

**Figure 1:**
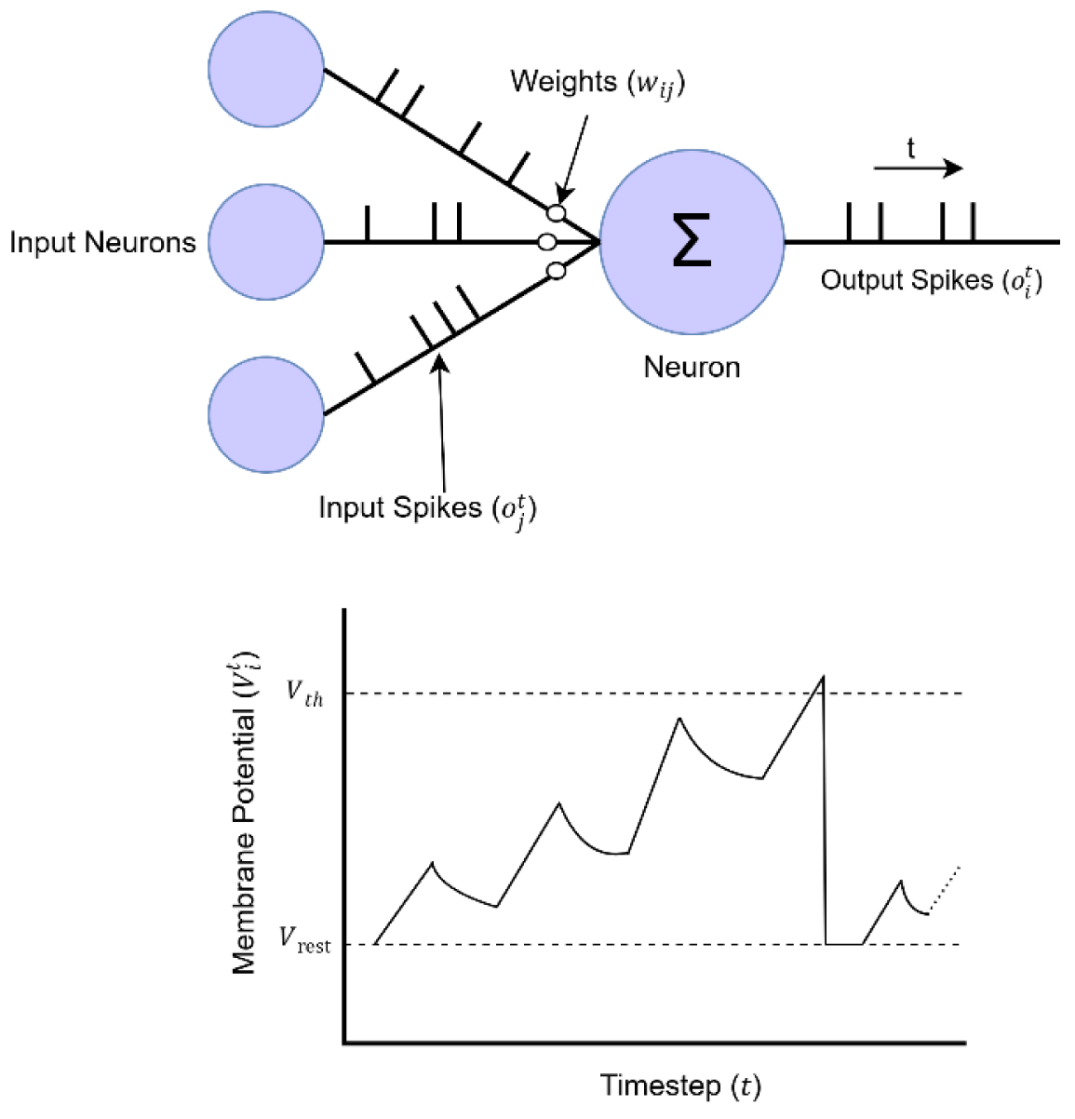
Leaky-integrate-and-fire neuron model.

Spiking Neural Networks (SNNs) are the third generation of ANN that was created to more accurately approximate the event-driven behavior of biological neurons. SNNs capture data by converting data into discrete spikes rather than by continuously valued activations and thus provide a way to efficiently encode and manipulate temporal and spatial information in a biologically plausible way. Because the events occurring in the networks are modelled as discrete spikes, the power consumption of SNNs is very low. Consequently, SNNs are particularly suitable for neuromorphic hardware and edge computing (Wang et al., 2024). The spiking neuron is the basis of SNN and encodes the temporal dynamics of the membrane potential of the biological neuron (Nunes et al., 2022). The Leaky Integrate-and-Fire (LIF) model is the simplest and most frequently used abstraction of the spiking neuron (Venkatesha et al., 2021), and it can describe the dynamics of a biological neuron by analytically analyzing the dynamics of the membrane potential as a function of V(t). Figure 1 illustrates the LIF model. For this purpose, the LIF neuron accumulates the incoming synaptic currents in the form of current spikes, and its dynamic behavior is described by the following differential equation:


$$\tau_{m}\frac{\mathrm{dV}(t)}{\mathrm{dt}}=-(V(t)-V_{\text{rest}})+R_{m}I(t)$$

Where *V (t)* represents the membrane potential at time *t*, *V*_*res*t_ is the resting potential of the neuron, *R_m_* is the membrane resistance, *I(t)* is the input current received from presynaptic neurons, and τ*_m_* - *R_m_C_m_* is the membrane time constant, where *C_m_* is the membrane capacitance. The negative term on the right-hand side represents the leakage effect, which causes the membrane potential to decay toward its resting state in the absence of input. This makes the LIF model more biologically realistic compared to the simpler integrate and fire model, which assumes perfect integration without decay (Nunes et al., 2022).

When the membrane potential reaches a certain threshold *V_th_*, the neuron emits a spike, and the potential is reset to a predefined value *V_reset_*, which is the resting potential:


$$V(t)=\{\begin{array}{c} V_{\text{reset},\;}\text{if}\;\;V(t)\geq V_{\mathrm{th}}\\ V(t),\;\;\;\text{otherwise}\;\;\; \end{array}$$

It is thus consistent with the way biological neurons generate action potentials based on the arrival of a level of depolarization. Computing differential equations by continuous methods is computationally expensive and inefficient, especially for high-dimensional or highly complex systems (Butcher, 2016). Hence it is usually discretized to be able to use in simulations and neuromorphic systems (Mehonic et al., 2020). The discrete-time LIF neuron in which the membrane potential is updated at discrete timesteps is given by:


$$V_{i}^{t}=\lambda V_{i}^{t-1}+\sum_{j}w_{\mathrm{ij}}O_{j}^{t-1}$$

where *V_i_^t^* is the membrane potential of neuron *i* at time step *t*, λ is a decay factor (0 < λ < 1) representing leakage, *w_ij_* is the synaptic weight from presynaptic neuron *j* to neuron *i*, and *o_j_^t − 1^* is the spike output of neuron *j* at the previous timestep (Xie et al., 2022). This equation efficiently models how the neuron integrates weighted inputs while accounting for membrane potential decay, making it more computationally tractable than solving the differential form.

A neuron generates a spike when its membrane potential exceeds the threshold Vth, which can be expressed mathematically as:


$$o_{i}^{t}=\{\begin{array}{c} 1,\text{if}\;\;V_{i}^{t}\geq V_{\mathrm{th}}\\ 0,\text{otherwis}e \end{array}$$

where *o^t^_i_* is the binary spike output (Rathi et al., 2020). However, this function introduces a major difficulty in training SNNs using traditional gradient-based methods, as the derivative of a step function is zero almost everywhere and undefined at the threshold (Rathi et al., 2020).

To address this problem, surrogate gradient methods apply a smooth function to approximate the derivative of the threshold function (Neftci et al., 2019). However, backpropagation can also be applied to SNNs without surrogate gradient approximations; event-based methods have been shown to compute exact gradients directly (Wunderlich and Pehle, 2021). Beyond backpropagation, pure Hebbian learning rules offer an alternative training paradigm for SNNs (Kempter et al., 1999), though for such learning to be effective the network must initially reside in an appropriate dynamical regime, such as near a critical point (Das and Levina, 2019). A very widely used approximation is represented as a piecewise linear function:


$$\frac{\partial o_{i}^{t}}{\partial V_{i}^{t}}=\xi \max(0,1-\frac{|V_{i}^{t}-V_{\mathrm{th}}|}{V_{\mathrm{th}}})$$

where *ξ* is a scaling factor that controls the gradient flow. This allows the use of backpropagation-like techniques for training SNNs while preserving the spike-based nature of the computation.

SNNs have several advantages in terms of event-based computation that allows them to be executed on neuromorphic hardware, where their spike-based sparsity translates into significant energy efficiency gains. Examples of such devices are IBM’s TrueNorth, Intel’s Loihi and the SpiNNaker system. These systems utilize the sparseness and asynchronous nature of spike-based computation to provide low power real-time processing (Shrestha et al., 2022).

Despite the above-mentioned advantages, SNNs currently lack integration with conventional deep learning architectures. However, in the future, with advanced research and techniques, SNNs remain a promising avenue for artificial intelligence.

### 2.2 Federated learning (FL)

Federated Learning (FL) is a decentralized machine learning methodology that permits several dispersed devices to work together to train a global model without exchanging raw data to protect data privacy (Zhang C. et al., 2021). FL enables local models to be trained on edge devices without centralizing sensitive data, and the model updates are sent to a central server for aggregated learning. It is helpful in application fields including healthcare, banking, and autonomous systems since it guarantees data privacy preservation and lowers the chance of data leaking.

Federated learning is very useful for edge devices as the constraints that they face are intrinsically limited (Imteaj et al., 2022). Centralized learning methods like deep learning require a large amount of raw data to be uploaded to a central server that may be unsuitable for edge applications on many vital aspects like privacy, bandwidth and latency. To reduce communication overhead and ensure that there is no downtime but local data storage on the edge devices, the federated learning approach makes use of the support for on-device learning, where the models are trained locally at the edge device and only the updates are transmitted to the server (Abreha et al., 2022). This is essential for applications such as the Internet of Things (IoT), Self Driving Cars, Mobile Health and Smart Grids in which the data processing must be done real time locally on the edge so that it can inform future decisions. The idea of Federated Learning can be used in an iterative model where a subset of clients learns a local model from their data and the server aggregates those using Federated Averaging (FedAvg) to update the global model (Sun et al., 2023). Federated Learning is highly useful, although there are several problems with Federated Learning like the non-IID data, latency in communication, resource limitations in edge devices and limited bandwidth (Zhang et al., 2022). The challenges include achieving efficient model compression, adaptive aggregation algorithms, and optimization protocol to ensure scalability and secure training.

The combination of the technique with neural network models designed for power-efficient operation on neuromorphic hardware, including SNNs, has been the primary focus of the current federated learning (FL) literature to enable low-latency inference on edge devices (Venkatesha et al., 2021). An edge artificial intelligence (AI) platform may learn in real time from decentralized data repositories, adjust to a changing environment, and customize the model while maintaining system performance and user privacy.

### 2.3 Audio signal processing

For a variety of applications, including smart appliances, security, health monitoring, and voice recognition, audio signal processing is required for the analysis and restoration of acoustic properties. The field looks into ways to turn unprocessed audio signals into insightful descriptions so that outliers can be effectively detected, improved, and classified.

Traditional techniques used in audio processing are Fourier transform-based, which extract the frequency contents of the signals and thereby obtain useful information about their spectral properties (Umapathy et al., 2010). Deep learning methods contribute to this by finding hidden patterns in large numbers of data sets which improves the accuracy in classification and anomaly detection applications (Purwins et al., 2019). A widely used representation of the audio primarily signals are spectrograms which are used to visualize audio in the frequency domain, aiding in the investigation of sound patterns.

Mel-spectrograms are a type of perceptually scaled representation of spectrograms, whose frequency inputs are converted into the Mel scale (Zhang T. et al., 2021). These representations are regularly used in deep learning models of audio and speech processing since they provide image representations of important auditory attributes with low computational cost. Short Time Fourier Transforms (STFT) spectrograms on the other hand preserve a high frequency resolution over time and thus they can be used in applications such as acoustic event detection and speech analysis (Parchami et al., 2016). Fourier Transforms (STFT) spectrograms on the other hand preserve a high frequency resolution over time and thus they can be used in applications such as acoustic event detection and speech analysis (Parchami et al., 2016).

An explicit representation of a Constant-Q Transform (CQT) spectrogram is given to attain greater resolution at lower frequencies (Yang and Das, 2019). CQT spectrograms can be utilized for music analysis if the harmonic structure of the sound is of great importance since frequency scaling takes the form of logarithmic frequency modulation. According to reports, wavelet spectrograms are also effective at identifying transient and non-stationary sounds, which satisfies the objective in biomedicine and environmental sound categorization (Dwivedi et al., 2019).

In this study, SNN and federated learning have been combined with audio signal processing to create a novel architecture for decentralized artificial intelligence models with competitive accuracy on resource-constrained edge devices. Federated learning allows for distributed training of SNN-focused models on edge devices in terms of data privacy and is an ideally relevant model in real time audio anomaly detection and personalized healthcare monitoring. Event driven processing can be done with low computational complexity and may lead to better performance on edge devices.

## 3 Methodology

This section introduces the federated learning process adopted for SNNs and gives an overview of the system architecture, model aggregation scheme, training and data distribution and processing.

### 3.1 System architecture

**Figure 2:**
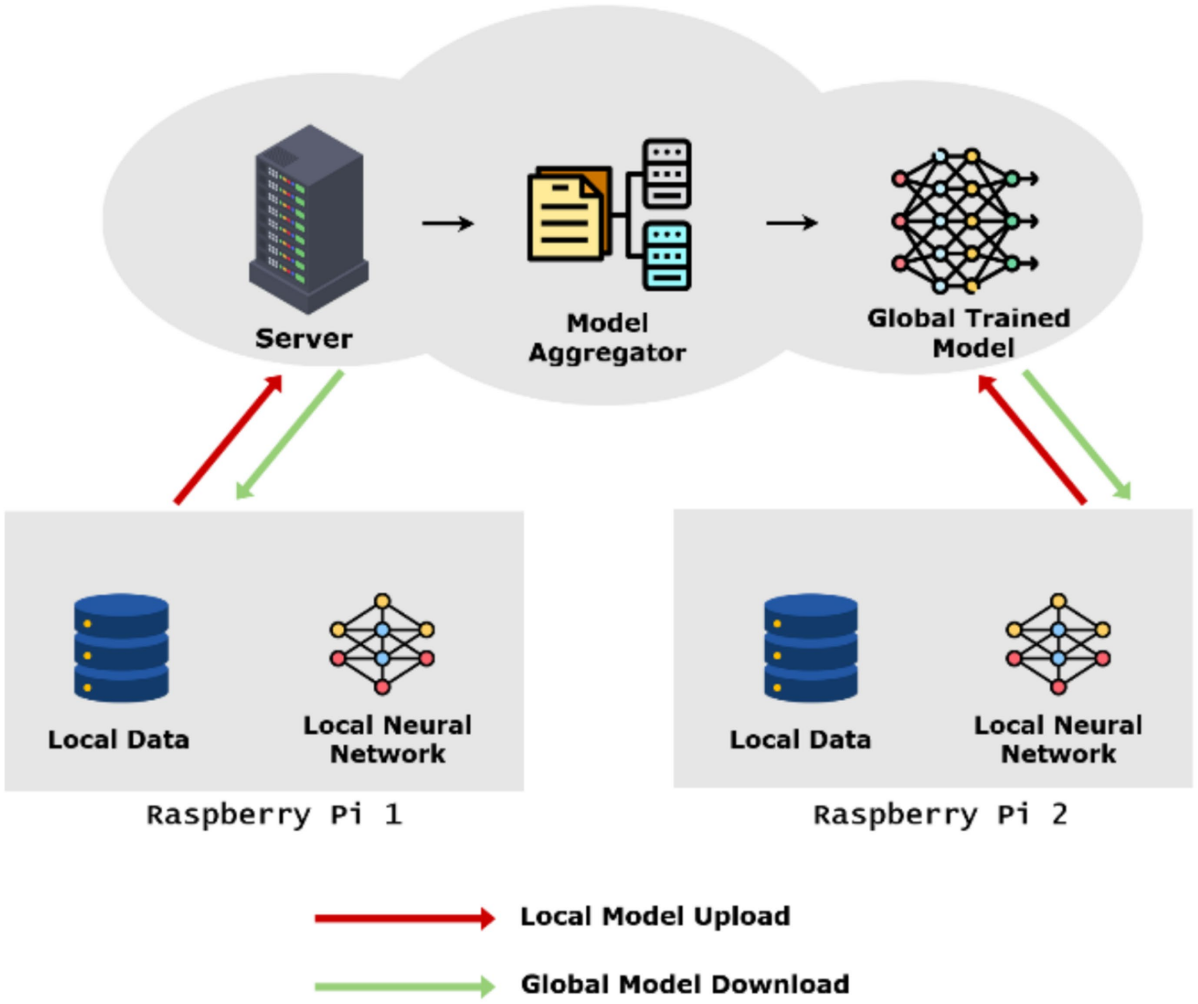
System architecture diagram.

In a federated learning framework with a server–client model, the client devices use local training of a SNN model from their respective data and thus promote decentralization and raw data privacy (Pfeiffer and Pfeil, 2018). After local training, each client device transmits its updated model parameters to the central server where the updated model parameters are averaged via an update mechanism to form a global model (McMahan et al., 2016). The collaborative architecture allows the global model to be trained on a wide range of data with respect to the properties of each user (Kairouz et al., 2019). In addition, the architecture distributes the computational workload local to the clients in an efficient and scalable manner (Li et al., 2020). Further, the configuration avoids constant transfer of data to each client, hence reducing communication overhead while still maintaining the effectiveness of model updates. Future iterations of the system can incorporate improved aggregating techniques and communication protocols. The global model can expand over time to consider new data patterns and emerging conditions thanks to regular model updates (Abhishek et al., 2022). The proposed system architecture diagram is shown in Figure 2.

### 3.2 Spiking neural network model

The model is a Convolutional Neural Network (CNN) that employs spiking neuron dynamics to provide improved spatial and temporal feature extraction. An input layer receives the raw data as spectrograms and is then processed via a series of convolutional blocks. The first block is a convolutional layer that converts the input into 16 feature maps composed of a 33 kernel with stride corresponding to the spatial dimensions and appropriate padding, which preserves the spatial dimensions. Batch normalization is then carried out to help control the learning process and max pooling is performed to optimize the spatial resolution. A spiking neuron layer which uses LIF dynamic with decay factor 0.95 adds a temporal dimension to feature extraction.

In the second block, the network further processes the data by increasing the depth from 16 channels to 32 channels using a similar convolutional operation. This is followed by batch normalization and max pooling, wherein the spiking neuron layer applies LIF dynamic with respect to the data over time. In the third convolutional block, the network again increases the channel count from 32 to 64, followed by batch normalization and max pooling, and implements temporal dynamics with respect to the data over time.

After the convolutional and spiking neuron blocks, the network flattens the feature maps with their output into a one-dimensional vector, which is then passed through a fully connected layer which reduces the dimension to 128 neurons. After applying dropout layer, the data has been performed with a probability of 0.5, to stop overfitting, before another spiking neuron layer is conducted to further improve temporal dynamics. The final fully connected layer maps the features to the number of target classes, to produce the output logits. The entire architecture can be applied to multiple discrete time steps. The temporal dynamics of the spiking neuron helps the network capture the evolution of features across these time steps, to create a useful approach can be presented for temporal classification tasks.

While convolutional layers operating on spectrograms can capture temporal patterns as spatial correlations along the time axis, they process the entire temporal window simultaneously and treat all features equally regardless of their persistence. The interleaved LIF spiking neuron layers address this by introducing temporal filtering through membrane potential dynamics: each neuron accumulates evidence across discrete timesteps and only fires when sufficient activation persists, naturally suppressing transient noise while amplifying sustained patterns. For respiratory audio classification, where distinctions between conditions depend on temporal properties of acoustic events such as the short, discontinuous nature of crackles versus the sustained nature of wheezes, this spike-based filtering provides a biologically motivated alternative to adding recurrent layers or temporal attention mechanisms to achieve similar functionality.

### 3.3 Federated training and model aggregation

In the current study, the dataset contains eight different classes: Healthy, LRTI (Lower Respiratory Tract Infection), URTI (Upper Respiratory Tract Infection), Pneumonia, Asthma, Bronchiectasis, Bronchiolitis, and COPD (Chronic Obstructive Pulmonary Disease), with each class in a different directory (Rocha et al., 2019). The augmented dataset is provided to both Raspberry Pi (RPI) clients. By using Dirichlet splitting, the data distribution assigned to each client is modified in a manner such that each client observes different segments of the data in the same dataset (Li et al., 2022). This method promotes heterogeneity in the local datasets, which is very important in measuring the robustness of the federated learning system under non-identically distributed (non-IID) conditions.

After local training on each RPI, the global model is updated using the Federated Averaging (*FedAvg*) algorithm (Sun et al., 2023). At round *r*, each client *c* trains a local model *M_c_* on its dataset *D_c_* for *K* epochs and sends the updated parameters back to the central server. The global model *M_r + 1_* is then computed via weighted averaging:


$$M_{r+1}=\frac{1}{\sum_{c\in P}|D_{c}|}\sum_{c\in P}|D_{c}|M_{c},$$

where *P* denotes the set of all participating clients and *|D_c_|* represents the number of samples on client *c*.

In addition to FedAvg, other types of federated averaging are implemented to contrast the optimal trade-off between convergence rate, stability against non-IID data, and communication efficiency in a heterogeneous federated learning scenario.

The first implementation, FedAdam, is an enhancement of FedAvg by including adaptive moment estimation (Reddi et al., 2020). In this case, the server has a first moment *m_r_* and second moment *v_r_* for the aggregated update *∆M_r_,* which is calculated as


$$\Delta M_{r}=\frac{1}{\sum_{c\in P}|D_{c}|}\sum_{c\in P}|D_{c}|(M_{c}-M_{r}).$$

The moment estimates are updated according to


$$m_{r+1}=\beta_{1}m_{r}+(1-\beta_{1})\Delta M_{r},$$

$$v_{r+1}=\beta_{2}v_{r}+(1-\beta_{2}){\left(\Delta M_{r}\right)}^{2}.$$

and the global model update is given by


$$M_{r+1}=M_{r}-\eta \frac{m_{r+1}}{\sqrt{v_{r+1}}+\epsilon }$$

with $\beta_{1}$ and $\beta_{2}$ being the decay rates, η the learning rate, and ϵ a small constant for numerical stability.

Another extension, FedPAQ, incorporates periodic averaging and quantization to reduce communication overhead. In FedPAQ, each client performs multiple local updates steps and computes a local update *Δ* $M_{c}$ . The update is then compressed using a quantization operator Q(·), and the quantized update *Q*(Δ $M_{c}$ ) is sent to the server, which then updates the global model as follows:


$$M_{r+1}=M_{r}+\frac{1}{|P|}\sum_{c\in P}Q(\Delta M_{c})$$

The fourth approach, known as Top K FedAvg, optimizes communication efficiency by transmitting only the top components of the update (Sattler et al., 2020). Based on this approach, each client takes the top K components (in terms of absolute value) from its update Δ $M_{c}$ , and they are denoted as $\Delta M_{c}^{\text{topK}}$ . The server goes ahead and updates the global model with the summation of the compressed updates.


$$M_{r+1}=M_{r}+\frac{1}{|P|}\sum_{c\in P}\Delta M_{c}^{\text{topK}}$$

These averaging methods were chosen because they offer complimentary advantages in tackling the inherent difficulties that are a fundamental component of federated learning. A useful baseline that uses weighted aggregate to capture the proportionality of each client’s contribution is FedAvg. By employing moment estimation to modify learning rates, FedAdam enhances the baseline, facilitating stabilization and accelerating convergence in diverse data environments. FedPAQ effectively lowers communication costs by taking advantage of periodic updates and quantization, both of which are important in low-bandwidth environments. Lastly, Top-*K* FedAvg ensures efficiency without significantly sacrificing model accuracy by concentrating on the most important updates and reducing the amount of data conveyed.

### 3.4 Local training on edge devices

The local SNN model is updated utilizing the locally stored data sets that each device possesses in a decentralized manner for local device training. To ensure consistency, the training and test datasets are separated using designated deterministic techniques. More realistic data splits can also be achieved by Dirichlet splitting (Reisizadeh et al., 2019). Data distribution across devices is on a non-IID (Non-Independent and Identically Distributed) basis, which simulates the heterogeneity that can exist in real life. Also, the system supports heterogeneous training on devices, with provisions for variable features of the data where each device will run a normal training sequence. Cross-entropy loss and the Adam optimizer are used to update the parameters of the model over several epochs. Both PyTorch and snntorch are used in the training sequence to do efficient floating-point computations (and spiking neuron dynamics) (Eshraghian et al., 2023). The new weights of the model are sent to a central server following local training by a socket-based connection. This architecture reduces redundant transfer of data and uses the computational power of edge devices to increase overall efficiency and data privacy.

The purpose of the snntorch library is to allow easy integration of SNNs in PyTorch. Traditional ANNs are designed to use continuous valued activation while SNNs operate by exploiting discrete spikes that occur temporally. Snntorch provides a set of tools for representing, deriving and learning spiking neuron models in addition to those two well-known and frequently studied spiking neuron models: Leaky Integrate-and-Fire (LIF) neurons and Integrate-and-Fire (IF) neuron models which both model temporal dynamics by upstream membrane potential fluctuations and spike generation mechanisms.

In SNNs training with the snntorch library, special techniques are needed to compensate for the non-differentiability of spike events (Eshraghian et al., 2023). So, to train efficiently, we use surrogate gradient methods that estimate the gradient of the spiking function so that backpropagation can be used in the model. Our model trains in discrete time steps and the input data are presented as spike trains. This requires using rate coding or temporal coding. The snntorch library is fully compatible with PyTorch’s autograd feature and can be used to compute gradients faster and update weights using optimization algorithms (like Adam). The library works with batch processing and uses GPU acceleration, so the library can be scaled across various hardware configurations.

To update the global model in the fewest possible steps without displaying raw data, we present a decentralized training pipeline in this work that, in terms of computational efficiency, approximates the dynamics of spiking neurons. The pipeline then projects to a central server where all the updated model weights are aggregated.

### 3.5 Dataset and spectrogram-based feature extraction

**Figure 3:**
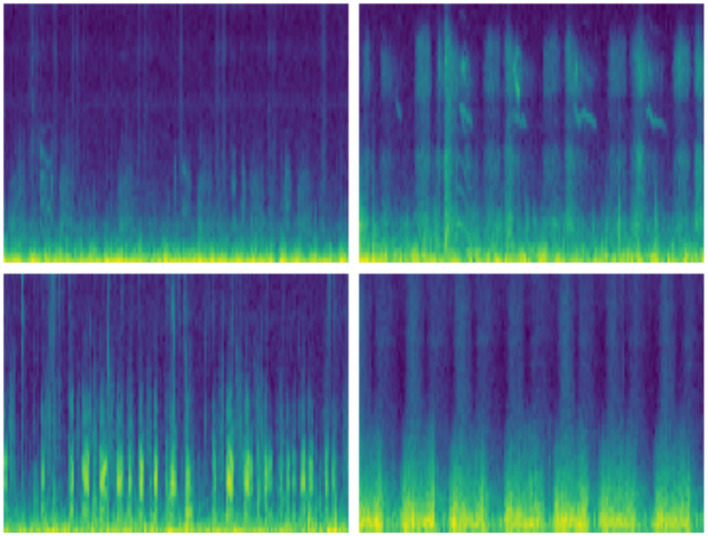
Spectrograms of four different respiratory audio samples.

This study uses the ICBHI Respiratory Sound Dataset, which was made available for the first time as a challenge in the 2017 International Conference on Biomedical and Health Informatics (ICBHI) (Rocha et al., 2019). It is a dataset containing respiratory sound recordings from a variety of patient populations with diverse respiratory conditions and clinical settings. Both the range of labels provided in the dataset and the common use of respiratory sound data in the respiratory sound community make it an appropriate parameter for similar tasks such as detection and classification of diseased sounds (Kim et al., 2021). Figure 3 represents spectrograms of different respiratory audio samples.

The provided part of the ICBHI dataset contains 920 audio segments. They are converted into spectrogram images using the temporal-frequency features that comprise every recording. The 920 spectrogram images distributed among the eight classes are as follows:

- Healthy
- LRTI (Lower Respiratory Tract Infection)
- URTI (Upper Respiratory Tract Infection)
- Pneumonia
- Asthma
- Bronchiectasis
- Bronchiolitis
- COPD (Chronic Obstructive Pulmonary Disease)

As data augmentation approach Vocal Tract Length Perturbation (VTLP) was employed to increase the diversity of the audio dataset (Kim et al., 2019). VTLP implements frequency warping directly onto the mel spectrograms generated from the audio signals. The frequency warping is governed by the relationship:


$$f^{\prime }=\mathrm{\alpha f}$$

where *f′* is the warped frequency and f is the original frequency. The warping factor *α* is typically chosen from a range, such as 0.8 ≤ *α* ≤ 1.2, to create realistic variations in vocal tract length. By varying *α*, we can simulate different vocal tract lengths, which helps in improving the model’s robustness to user variability.

Following augmentation, the dataset was balanced to approximately 180 spectrograms per class across all eight categories, yielding a total of 1,440 samples. For the dominant COPD class, balanced subsampling was applied prior to augmentation to prevent over-representation, while the remaining classes were augmented within a controlled ratio to preserve sample diversity. This balanced configuration ensures that model performance metrics reflect genuine learning across all classes rather than majority-class bias, and that the per-client data partitions produced by Dirichlet splitting retain meaningful class representation even at low concentration values.

Although designed for use in voice, it gave notable results when applied to the respiratory dataset (Nguyen and Pernkopf, 2022). Using VTLP not only increases the amount of training data we have, but it also introduces variability into our spectrograms, which improves model generalization by accounting for different users and improving classification results.

## 4 Experimentation and results

This section provides an overview of the proposed federated learning architecture for SNNs as well as experimental configuration, performance metrics and comparison of various configurations.

### 4.1 Hardware and communication setup

The federated learning system is installed on a central computer as the server and two Raspberry Pi 5 single board computers as the client nodes. The Raspberry Pi 5 boards are powered by a quad-core Arm Cortex-A76 processor with a frequency of up to 2.4 GHz and backed by 8GB of LPDDR4X-4267 RAM, offering significant processing capabilities compared to its predecessors. All devices are connected to a central Wi-Fi network with considerable speed and reliability. The training protocol is designed such that the Raspberry P is perform local training sequentially, and the updated model weights and biases are sent wirelessly to the central server after each training cycle for aggregation. Since the updates are only made at discrete time and not in real-time, the speed of the network, while sufficient, does not present a strong bottleneck in this arrangement.

Preliminary attempts were made to measure power consumption using software-based monitoring tools and inline power sensors on the Raspberry Pi 5. However, these measurements proved unreliable for drawing meaningful comparisons between SNN and CNN workloads. Software-based power estimation on ARM processors lacks the granularity to isolate model-level power draw from system-level overhead, and consumer-grade inline sensors introduced sufficient noise to render the resulting power-over-time profiles inconclusive. Furthermore, the SNN training was executed using snnTorch on a Von Neumann architecture, where spike-based operations are simulated through conventional floating-point arithmetic rather than native event-driven hardware. This means the power profile reflects PyTorch’s general-purpose tensor operations rather than the sparse, asynchronous computation that would occur on neuromorphic silicon. Accurate power characterisation of federated SNN training would require either instrumentation-grade power monitoring equipment or deployment on neuromorphic hardware where the energy advantages of spike-based computation can be directly observed. We consider this an essential direction for future work.

### 4.2 Baseline models

The baseline CNN architecture consists of an input layer to the network which is represented as a single-channel image and consists of three convolutional blocks. The first convolutional block comprises 16 filters. These filters represent the basic features, which are normalized using batch normalization and downsampled using max pooling. The next block doubles the feature depth to 32 filters using the same convolutional setup, batch normalization and max pooling. The third block then doubles the feature depth to 64 filters, normalizes and downsamples using max pooling.

After these the convolutional blocks feature maps are flattened into a one-dimensional vector. A fully connected layer is trained to process this vector to reduce overfitting. A second unsqueeze operation is performed to adjust the output dimensions. The proposed CNN architecture provides a tuned feature extraction and classification solution that performs efficiently and effectively in a distributed training environment. The model architecture also helps us compare the performance of SNNs and CNNs due to their similarities.

### 4.3 Training performance

Results of experiments, displayed in the tables, show that SNNs and CNNs perform well both in heterogeneous federated learning systems, pointing that SNNs are dependable in federated setup. All reported accuracy and Macro F1 values represent the mean across five independent runs with different random seeds. Standard deviations are reported alongside each metric to indicate result stability.

Table 1 presents baseline models in a single-client setting: the SNN with an accuracy of 97.19% and a Macro F1 score of 0.9738 which is very comparable to that of the CNN with an accuracy of 98.12% and a Macro F1 score of 0.9824 which supports the notion that SNNs can perform classifying tasks very well in the context of centralized systems. In spite of the slightly higher speed of convergence to 90% accuracy (at 3 rounds versus the SNN at 8 rounds), the competitiveness and the high accuracy of the SNN suggest the possibility that the SNN can be used in edge computing, particularly as neuromorphic edge hardware becomes more widely available.

**Table 1:** Federated training on a single-client baseline systems: SNN vs. CNN configurations.

Configuration	Accuracy (%)	Macro F1	Time-to-90% (rounds)
SNN – 1 Client	97.19 ± 0.84	0.9738 ± 0.0071	8
CNN – 1 Client	98.12 ± 0.53	0.9824 ± 0.0048	3

On each Raspberry Pi 5 client, the average wall-clock time per local training round was 47.3 ± 2.1 s for the CNN and 214.6 ± 5.8 s for the SNN. The SNN’s approximately 4.5 × training overhead relative to the CNN is attributable to the temporal unrolling across discrete timesteps required by the LIF neuron simulation and surrogate gradient backpropagation through time. This means that in wall-clock terms, the SNN’s 8-round convergence to 90% accuracy in the single-client baseline (Table 1) corresponds to approximately 28.6 min of local training time, compared to 2.4 min for the CNN’s 3-round convergence, a 12 times difference that the round-count metric alone does not capture.

**Figure 4:**
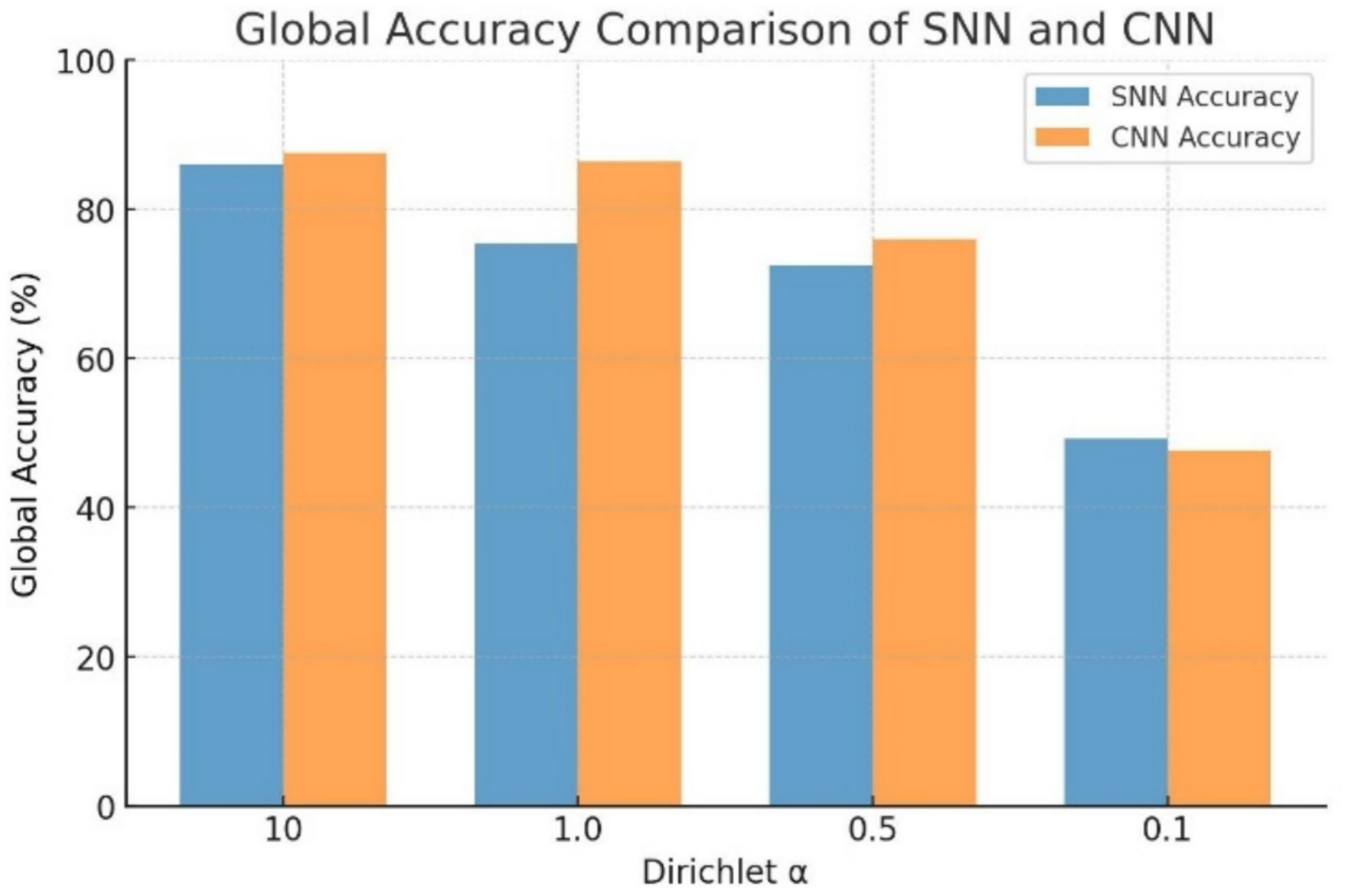
Global accuracy comparison of SNN and CNN.

**Figure 5:**
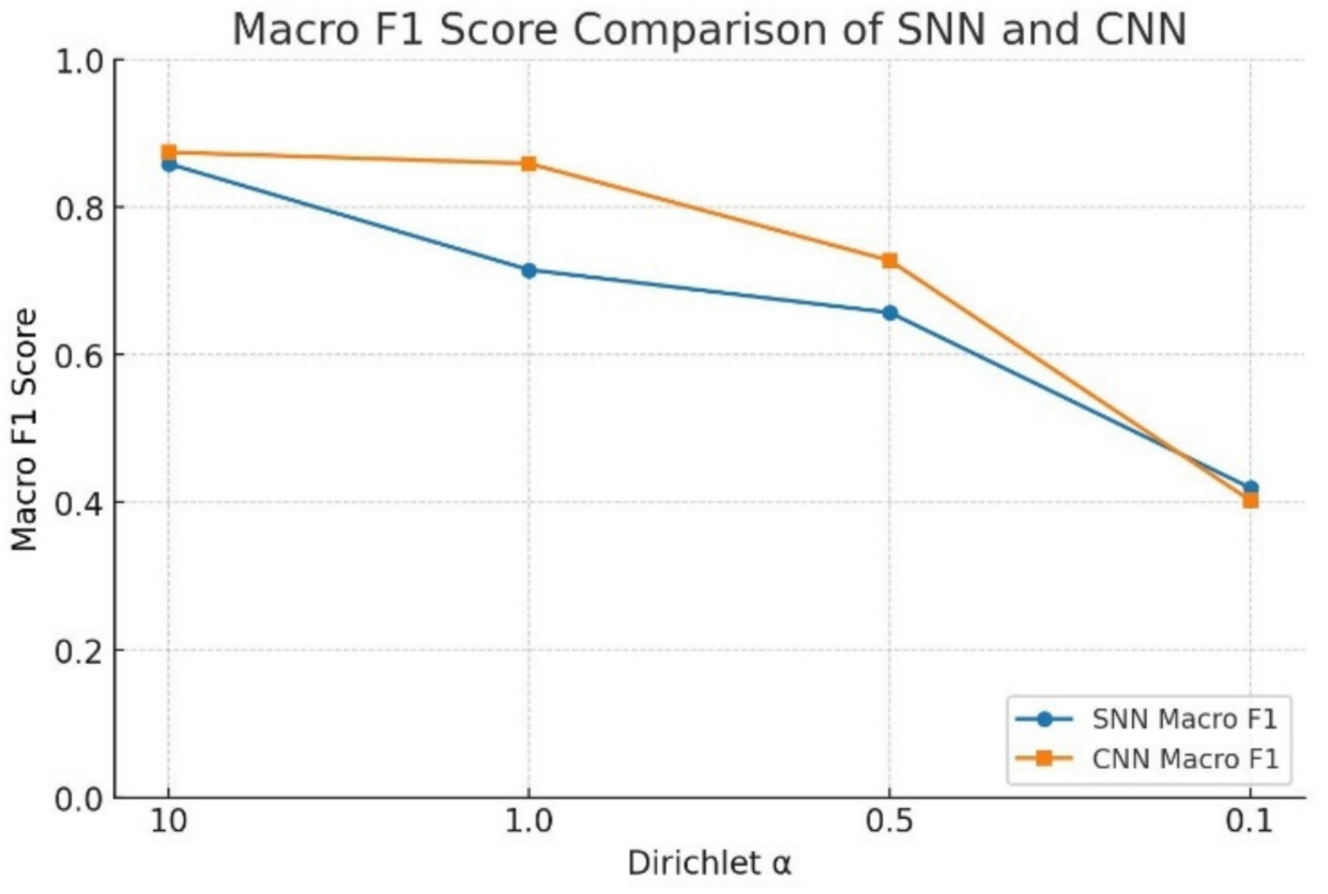
Macro F1 score comparison of SNN and CNN.

Table 2 shows the federated learning analysis under multi-client Dirichlet data splitting and FedAvg aggregation, in which the SNN model demonstrates robustness across different Dirichlet concentration parameters *α*. At *α* = 10, the SNN achieves a global accuracy of 85.94% and a Macro F1 score of 0.8588, which is comparable to the CNN’s accuracy of 87.50% and Macro F1 score of 0.8743. At *α* values of 0.5 and above, the CNN holds a modest performance advantage, but the SNN remains competitive, particularly at higher *α* values where data imbalances are less pronounced. At *α* = 0.5, the SNN still achieves an accuracy of 72.50%, demonstrating resilience under increasing data heterogeneity. The visual representations of these results are given in Figures 4, 5.

**Table 2:** Federated training on a two-client system: SNN vs. CNN configurations under Dirichlet splitting and FedAvg aggregation.

Model	Dirichlet *α*	Global accuracy (%)	Macro F1	Time-to-70% (rounds)
SNN	10	85.94 ± 1.42	0.8588 ± 0.0139	11
	1.0	75.31 ± 1.87	0.7151 ± 0.0216	15
	0.5	72.50 ± 2.76	0.6571 ± 0.0284	13
	0.1	49.19 ± 3.54	0.4196 ± 0.0397	–
CNN	10	87.50 ± 1.14	0.8743 ± 0.0108	3
	1.0	86.34 ± 1.38	0.8591 ± 0.0152	4
	0.5	75.94 ± 2.31	0.7277 ± 0.0247	4
	0.1	47.68 ± 3.92	0.4025 ± 0.0436	–

At *α* = 0.1, both models experience a severe accuracy collapse, with the SNN dropping to 49.19% and the CNN to 47.68%, representing a 36-point and 40-point decline from their respective α = 10 baselines. Despite this degradation, both models remain well above the 12.5% accuracy that a majority-class classifier would achieve on the balanced eight-class dataset, indicating that meaningful discriminative capacity persists even under extreme heterogeneity. However, the failure of both models to reach the 70% convergence threshold at *α* = 0.1, as reflected by the dash entries in Table 2, confirms that this degree of non-IID partitioning with only two clients exceeds the practical limits of FedAvg-based aggregation for both architectures. Notably, *α* = 0.1 is the only setting in which the SNN outperforms the CNN, reversing the pattern observed at all other concentration values. We attribute this reversal to the implicit regularization properties of spike-based computation. The binary firing threshold in LIF neurons naturally filters out weak or noisy activations that fall below threshold, whereas a CNN propagates all activations regardless of magnitude. Additionally, the surrogate gradient used during backpropagation is itself an approximation, which limits how precisely each client’s model can fit its local data distribution. Together, these two properties prevent the SNN from overfitting as aggressively to skewed local data as the CNN does, resulting in less weight divergence when updates are aggregated at the server. This is consistent with the noise tolerance of federated SNNs observed by Venkatesha et al. (2021) and suggests that the robustness advantage of SNNs may be most apparent in the extreme heterogeneity regimes where federated learning is most difficult.

Table 3 compares several aggregation methods for SNN training under a Dirichlet concentration of *α* = 0.5. FedAdam achieves the highest global accuracy of 89.06% and a Macro F1 score of 0.9022, outperforming FedAvg (72.50%) and FedPAQ (68.74%). This result demonstrates the value of adaptive optimization for SNN training under non-IID data distributions. The adaptive learning rate and momentum built into FedAdam allow it to compensate for the noisy and heterogeneous gradient signals that arise from combining surrogate gradient approximation with skewed client data, a compounding difficulty that is specific to federated SNN training. FedPAQ, which prioritises communication reduction through quantization and periodic averaging rather than gradient correction, lacks this adaptive capacity and consequently performs worst among the methods tested. Top-K FedAvg achieves accuracy on par with FedAvg (72.81%) while transmitting only the top 50% of update components, confirming that SNN weight updates contain sufficient redundancy for aggressive sparsification without additional accuracy loss. This sparsity tolerance aligns with the inherently sparse activation patterns of spike-based computation, suggesting that communication-efficient aggregation strategies may be naturally suited to federated SNN training.

**Table 3:** Comparison of different federated learning aggregation methods for SNN training with Dirichlet distribution concentration of α = 0.5.

Aggregation method	Global accuracy (%)	Macro F1	Time-to-70% (rounds)
FedAvg	72.50 ± 2.76	0.6571 ± 2.76	13
FedAdam	89.06 ± 1.38	0.9022 ± 0.0131	9
Top-K FedAvg	72.50 ± 2.63	0.6404 ± 0.0309	14
FedPAQ	68.74 ± 3.24	0.6286 ± 0.0341	–

Experimental results show that SNNs can be competitive and as reliable as CNNs for federated learning tasks. CNN is found to have slightly higher accuracy, even with decreasing convergence rate. But in comparison to CNN, performance for a federated learning task, SNNs show the closeness to CNNs in terms of performance which shows the reliability of SNNs in edge computing applications. We present additional empirical experiments demonstrating the robustness of SNNs to various degree of data heterogeneity. Here the federated learning experiments show the strength of SNNs against different degree of data heterogeneity including their high accuracy even in conditions with severe data distribution such as non-IID. The importance of adaptive optimization strategies is also demonstrated using an examination of several aggregation algorithms. FedAdam was found to be the best optimization strategy for improving SNN performance in non-IID scenarios.

## 5 Conclusion

In conclusion, the study demonstrates that SNNs can be a reliable alternative to traditional deep learning models like CNNs, especially in decentralized environments where robustness to data heterogeneity is a critical factor. The findings suggest that SNNs, when optimized with the right aggregation methods, can effectively handle heterogeneous data distributions, making them suitable for edge computing and federated learning applications. In edge scenarios such as IoT devices, wearable technology, and remote sensing systems, where computational resources and power consumption are constrained, the potential for energy-efficient deployment of SNNs on neuromorphic hardware makes them particularly advantageous as such processors become available for edge applications.

Future research could focus on optimizing training algorithms to improve convergence speed and accuracy while maintaining low power consumption. Expanding studies to include a larger number of clients and more diverse datasets would provide deeper insights into the scalability and generalizability of SNNs in federated learning settings. Additionally, investigating novel hardware accelerators and neuromorphic computing architectures tailored for SNNs could improve efficiency and scalability. Addressing security and privacy challenges in federated SNN training is also essential for ensuring robust deployment in sensitive applications such as healthcare and industrial automation.

## Data Availability

The original contributions presented in the study are included in the article/supplementary material, further inquiries can be directed to the corresponding author.
